# A Deep Learning-Based End-to-End Composite System for Hand Detection and Gesture Recognition

**DOI:** 10.3390/s19235282

**Published:** 2019-11-30

**Authors:** Adam Ahmed Qaid MOHAMMED, Jiancheng Lv, MD. Sajjatul Islam

**Affiliations:** College of Computer Science, Sichuan University, Chengdu 610065, China; adammohammed@stu.scu.edu.cn (A.A.Q.M.); sajjatulislam@stu.scu.edu.cn (M.S.I.)

**Keywords:** hand detection, hand gesture recognition, human-computer interaction, deep learning

## Abstract

Recent research on hand detection and gesture recognition has attracted increasing interest due to its broad range of potential applications, such as human-computer interaction, sign language recognition, hand action analysis, driver hand behavior monitoring, and virtual reality. In recent years, several approaches have been proposed with the aim of developing a robust algorithm which functions in complex and cluttered environments. Although several researchers have addressed this challenging problem, a robust system is still elusive. Therefore, we propose a deep learning-based architecture to jointly detect and classify hand gestures. In the proposed architecture, the whole image is passed through a one-stage dense object detector to extract hand regions, which, in turn, pass through a lightweight convolutional neural network (CNN) for hand gesture recognition. To evaluate our approach, we conducted extensive experiments on four publicly available datasets for hand detection, including the Oxford, 5-signers, EgoHands, and Indian classical dance (ICD) datasets, along with two hand gesture datasets with different gesture vocabularies for hand gesture recognition, namely, the LaRED and TinyHands datasets. Here, experimental results demonstrate that the proposed architecture is efficient and robust. In addition, it outperforms other approaches in both the hand detection and gesture classification tasks.

## 1. Introduction

Among the several human-computer research activities in computer vision and machine learning (e.g., human action recognition, pose estimation, and gesture recognition), hand gesture recognition is particularly important due to its various potential applications. Robust hand gesture detection and recognition in cluttered environments is a crucial task for many computer vision applications, such as human-computer interaction, sign language recognition, hand action analysis, driver hand behavior monitoring, and virtual reality. This task presents a challenging problem that has not yet been solved in computer vision and machine learning. Unlike many previous studies [1,2,3,4,5,6,7], which separately have tried to address hand detection or hand gesture recognition, our approach attempts to jointly solve the problem of hand localization and gesture recognition. This task, however, is very challenging, due to the significant variations of hand images in realistic scenarios.

The utilization of hand-crafted features has dominated early research in hand detection and gesture recognition. Most of these approaches have utilized hand skin color, texture, and appearance features for hand detection and gesture recognition [8,9,10,11,12,13]. However, their success is only found in certain well-prepared environments. The task remains challenging in real-life applications, due to problems posed by background complexity, occlusion, viewpoint, lighting changes, the deformable and articulated nature of hands, etc. Encouraged by recently emerged deep learning approaches for object detection and recognition [14,15,16], many researchers have proposed numerous approaches to deal with these problems. Despite the considerable degree of progress, these challenges still exist, even with current deep learning approaches [17,18].

In previous works, the study of hand gestures is broadly categorized into two groups, namely, static and dynamic hand gestures. Static hand gestures require only one image to convey meaningful information, whereas dynamic gestures involve a sequence of frames to perform one gesture. The focus of this work is to solve the problem of detecting and recognizing static hand gestures where the bare hand performs postures to disclose certain meanings. This problem, however, represents a high level of complexity, and retrieving the hand shape is difficult, due to the vast number of hand configurations and variations of the viewpoint with respect to the image sensor. Furthermore, recognizing static hand gestures plays an important role in many applications, such as sign language recognition for deaf and speech-impaired people [6,7], driver hand monitoring and hand gesture commands in order to reduce driver distraction [4,18], an alternative input method for interfacing between human and machines [2,8], in-air writing interaction [19], hand-object interaction in augmented and visual reality environments [20], and many other applications.

In this work, we have tried to address the problem of static hand gesture localization and recognition in two steps. The first stage of our proposed approach has addressed the accurate location and has extracted the hand from the given image by generating bounding boxes. This step requires sufficient data to build a robust model. Therefore, we have collected a large hand detection dataset that contains hands in various complex and cluttered environments. In the second stage, the gesture recognition has been performed from a predefined gestural vocabulary. For the first part of the proposed approach, we have trained a focal loss one-stage dense object detector to detect hands, namely, RetinaNet [21]. For the second part, we have proposed a lightweight convolutional neural network (CNN) based on MobileNet [22] for gesture recognition. The choice of a lightweight CNN was made to reduce the computational cost during the inference process, since it has been designed to work faster, for use, for instance, in embedded systems and resource constrained devices [23]. The training of the proposed architecture follows a stage-wise approach. We started the experiment by training the hand detector using our collected dataset to build a reliable detector. Then, we used the trained detector to extract hands from the gestural datasets and train the lightweight CNN architecture to recognize hand gestures. During the inference procedure, the architecture takes an input image with an arbitrary size and passes it through a hand detector to extract the hand region, which, in turn, passes through a lightweight convolutional neural network for hand gesture recognition. The output of the architecture is an image that contains the detected hand with a bounding box and a detection score, as well as the recognized hand gesture.

The contributions are summarized below in six points:

- We have designed an end-to-end deep learning-based architecture to jointly detect and recognize static hand gestures.
- This is the first approach using focal loss-based one-stage dense object detection for the purpose of hand detection. This method achieves a high degree of accuracy for hand detection in complex and cluttered environments, such as extreme illumination changes, low-resolution images, hand variations, high levels of occlusion, and variation in shape and viewpoint.
- The proposed approach does not require any preprocessing step, including image enhancement, hands at a specific distance from the camera, or any postprocessing step.
- The approach achieves state-of-the-art performance on various publicly available benchmarks for both the hand detection and gesture recognition tasks. The proposed system was evaluated on various standard hand datasets with varying degrees of complexity in terms of the clutter environment. The approach was tested on various gestural datasets with different vocabulary sizes.
- Although there are many available datasets that were used to evaluate hand detection systems in the literature, they do not provide sufficient annotated data to train a deep learning-based hand detector. Therefore, we have collected and annotated a large dataset that includes over 41,000 hand instances to train our model.
- Finally, we have designed our system to be adaptive and be able to be retrained using different gestural datasets, making it flexible to potentially use again with another dataset containing a different hand gesture vocabulary. The gesture classification model is the only component that needs to be retrained, while the rest of the architecture stays untouched.

The rest of this paper is structured as follows. Section 2 gives a brief review of some related works concerning hand detection and gesture recognition. Section 3 provides a detailed description of the proposed architecture and an elaboration on the function of all of its components. In Section 4, the description of the datasets, the evaluation metrics, and experimental setup used during evaluation, and comparison of the experimental results obtained are been presented. Finally, the conclusion of this work and directions for future work are presented in Section 5.

## 2. Related Works

The detection and recognition of hand gestures is inevitable for many human-computer interaction applications. Although many studies have focused on detecting hands or recognizing gestures separately, a robust system for the combined detection and interpretation of hand gestures has still yet been achieved. In this section, we provide a review of relevant literature concerning hand detection and gesture recognition methods using conventional handcrafted features, as well as those using deep learning networks.

### 2.1. Hand Detection

Many approaches have been proposed to address the constraints represented by hand detection. Those methods broadly fall into four categories, namely, skin segmentation, depth-based detection, hand detection based on hand-crafted features, and CNN-based approaches.

1. Skin color-based approaches: Traditionally, many algorithms for hand detection rely on skin color segmentation to detect and extract hands from the background. Dardas et al. [8] proposed a thresholding method to segment hands in the hue, saturation, and value (HSV) color space after extracting other skin regions, such as the face. Mittal et al. [24] used a skin-based detector to generate hand hypotheses for the first stage of their hand detection algorithm. To improve the robustness of skin segmentation, Stergiopoulou et al. [25] used a skin-probability map (SPM) in the HSV color space for skin color classification, along with extra information, such as motion and morphology weights of hands. To further enhance the skin segmentation, some hand-crafted features such as a Gabor filter, scale invariant feature transform (SIFT) and histogram of oriented gradients (HOG) were combined to segment the skin regions of hands [26]. Combining skin detection with deep learning object detectors has also been proposed. Roy et al. [5] proposed two architectures (patch-based CNN and regression-based CNN for skin segmentation). Their main purpose was to reduce the occurrence of false positives resulting from the estimated bounding boxes of recent object detectors [14,15]. However, skin segmentation-based hand detection is not robust enough in practice and suffers from several constraints, which include skin tone variation, occlusion, background clutter, poor illumination, etc.
2. Depth-based approaches: The recent development of emerging color-depth camera-based sensing techniques, such as the Microsoft Kinect^TM^, has solved many problems related to hand gesture recognition, including hand extraction using depth data [27]. A large portion of depth-based hand detection methods still rely on the distance between the hand and the sensor [1,2,3]. However, some studies attempted to exploit certain depth features for the per-pixel segmentation of hands. Keskin et al. [28] extracted scale invariant shape features from depth images then fed them into a per-pixel randomized decision forest (RDF) classifier. The final predicted label for the whole image was determined by majority voting. Kang et al. [20] proposed a two-stage random decision forest (RDF) method for detecting and segmenting hands. The first stage RDF attempted to locate the hand region from a depth map, while the second stage segmented hands in the pixel-level by applying the RDF method to the detected regions. Although those methods have succeeded in well-prepared indoor environments, the use of depth sensors might not be feasible in all environments, such those outdoors.
3. Hand-crafted features: Many hand-crafted features were also introduced to detect hands. Inspired by the success of Viola and Jone’s algorithm for face detection [29], which combines Haar-like features with an AdaBoost learning classifier, similar approaches have been proposed in [8,30,31]. Wang et al. [32] have used SIFT features and the AdaBoost learning algorithm to achieve in-plane rotation invariant hand detection. To speed up the testing process and boost accuracy, they proposed the use of the sharing feature concept. Despite its success in face detection, the framework of Viola and Jone’s was fragile when facing cluttered backgrounds, and the Haar-like features were not efficient enough to represent complex and articulate objects, such as human hands. Dalal and Triggs [33] proposed a gradient histogram feature descriptor called HOG for human detection. A variant of the HOG feature, called skin color histogram of oriented gradients (SCHOG), was proposed by Meng et al. [34] to construct a human hand detector. First, the SCHOG features were extracted by combining HOG with skin cues. Then, a support vector machine (SVM) algorithm was applied to construct a SVM-trained classifier for hand detection. Despite the improved results, the performance was still insufficient due to the large variations of hands’ appearances in unconstrained backgrounds. Mittal et al. [24] developed a two-stage hand detector. The first stage generates a hand proposal using three complementary detectors, namely, a skin-color-based detector, a deformable part model (DPM) based shape detector, and detectors with contextual cues (context detector). In the second stage, the scores of the proposals were combined and fed into a linear SVM classifier to compute the final prediction. Although the proposed detector achieved adequate precision performance, this method was computationally expensive (two minutes per image) and consequently is not feasible for real-time applications.
4. Deep feature-based methods: Inspired by the recent success of convolutional neural networks, researchers have proposed numerous methods for object detection and recognition based on CNNs [14,15,21,35]. Consequently, these methods have been developed and used for hand detection. Roy et al. [5] proposed a two-stage hand detector based on the region-CNN (R-CNN) and Faster R-CNN [14,15] frameworks. Initially, they used an object detection algorithm to generate hand regions and then a CNN-based skin segmentation was used to reduce occurrences of false positives during hand detection. Deng et al. [36] built a two-stage framework to jointly detect hands and estimate their orientation. The framework applied the region proposal networks (RPN) used in Faster R-CNN to generate region proposals, then estimated hand orientation based on the region of interest (ROI) pooling features. Furthermore, they claimed that the rotation estimation and classification could mutually benefit each other. Huang et al. [19] proposed an egocentric interaction system using Faster R-CNN [15] to locate and recognize static hand gestures. Their system achieved better performance on a challenging dataset under challenging conditions. Le et al. [4,37] introduced a novel approach that combined local and global context information to enhance the robustness of the deep features. They further extended the region-fully convolutional network (R-FCN) and Faster R-CNN by aggregating multiple scale feature maps. This approach achieved satisfactory performance on two challenging datasets. Although hand detection using CNN-based methods significantly improves the detection accuracy, it yields this good accuracy at a high computational expense. An efficient and fast algorithm is still required to robustly detect hands in unconstrained scenarios.

### 2.2. Hand Gesture Recognition

In recent years, researchers have proposed numerous approaches to ameliorate the recognition rate and speed of hand gesture recognition systems. Among the many successful methods for hand gesture recognition, a large portion still depend on hand-crafted features such Gabor filters [38], SIFT features [32], dynamic time warping (DTR) [2], Hu moments, and Zernike moments [9]. Among those methods, Dardas et al. [8] utilized Bag-of-Feature (BoF) descriptor to map SIFT features extracted from the hand region and fed them to SVM classifiers. Pisharady et al. [38] proposed a Bayesian model of visual attention, combining low level (color) and high level (shape, texture) features of the hand region, feeding them to a SVM for classification. Despite good accuracy, their method cannot be generalized to detect hands of arbitrary shapes.

Recently, deep learning-based methods have emerged and advanced the research in this area. Chevtchenko et al. [9] presented a novel approach based on combining traditional hand-crafted features with a CNN. They evaluated their approach on depth and grayscale images, where the background has already been removed using depth data by considering the hand as the closest object to the camera. Liang et al. [3] utilized CNNs as feature extractors from point clouds captured by a depth sensor. Their framework uses CNNs to extract features from view images projected from a point cloud. Finally, an SVM was trained for hand gesture recognition. Oyedotun and Khashman [39] proposed to use convolutional neural networks and stacked denoising autoencoders (SDAEs) to recognize 24 ASL (American Sign Language) hand gestures. Li et al. [17] proposed a soft attention mechanism to automatically localize and classify gestures using a single network. A sliding window approach was used to generate proposals, and then a pre-trained CNN architecture was used to extract image features. The attention network was applied to acquire the weight of each proposal to locate hands, and finally, a softmax function layer was used for gesture recognition.

Despite the success of the abovementioned methods, there is still a lack of a highly accurate approach to recognize hands in uncontrolled scenarios, such as those where hands are subjected to high occlusion, noise, poor illumination, etc. In this paper, we attempt to address these challenging problems by proposing a deep learning-based system to localize hands and recognize hand gestures under both real-life and uncontrolled situations. The proposed system uses a robust one-stage hand detector to localize hands in the input image. Then, a lightweight CNN network is responsible for classifying gestures.

## 3. Proposed System

**Figure 1:**
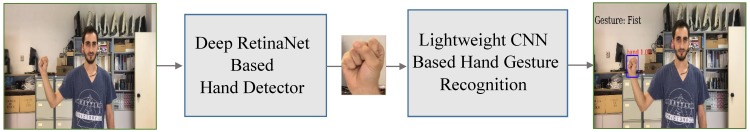
The general workflow of the proposed method for hand detection and gesture recognition.

As depicted in Figure 1, the overall architecture of the proposed system for hand detection and gesture recognition is illustrated. The captured image is passed through a RetinaNet-based hand detector to extract hand regions. Once the hand is detected, the hand region is then extracted and passed through a lightweight CNN for hand gesture recognition.

### 3.1. RetinaNet-Based Hand Detection

The architectures of recent object detection networks are usually divided into two categories, namely, one-stage and two-stage object detectors. In two-stage object detectors, like region-based CNNs [14,15,35], the first stage generates a sparse set of region of interest (ROIs) proposals, which should contain all objects while filtering out most of the negative locations, and the second stage classifies the proposals into foreground or background classes. In spite of the slow inference speeds, these two-stage detectors have dominated over the last few years due to their high detection accuracy, which results from their capability of maintaining an amendable balance between the foreground and the background. Over the years, numerous improvements have been proposed for single-stage detectors in terms of their inference speed [35] and by proposing learned object proposals such as region proposal networks (RPN) to generate the ROI proposals [15].

On the other hand, one-stage (or single-stage) object detectors (e.g., single shot multibox detection—SSD, [40] and You Only Look Once—YOLO [41]) have been designed for speed, but their detection accuracy is considerably less than that of two-stage methods [23]. These one-stage detectors skip the region proposal stage for the detection of foreground candidates and run both tasks simultaneously using a dense sampling of possible locations. Unlike two-stage detectors, which generate 2000 region proposals [15], one-stage detectors evaluate 10^4^–10^5^ region proposals per image, but only a few locations contain objects in reality. This issue is known as the class imbalance. The presence of a large number of easily-identified negative samples (in our case, non-hand objects) may overwhelm the detector. The training process of dense detectors becomes inefficient, as it is dominated by easily classified background examples. To overcome the problem of class imbalance encountered during the training of one-stage dense detector and thus improve the detection performance so that it outperforms two-stage detectors, focal loss [21] is proposed to alleviate easy negatives that do not contain interesting information and concentrate more on hard negatives. Therefore, our proposed solution is to modify the loss function to down-weigh easy examples and focus training on hard negatives.

The normal cross entropy (*CE*) loss for binary classification is defined as:

(1) $$CE\left(p,\;y\right)=\{\begin{array}{ll} -\log\left(p\right), & if\;y=1\\ -\log\left(1-p\right), & otherwise \end{array}\;\;$$

where y∈{±1} defines the ground truth class, and p∈[0,1] is the model’s estimated probability for the class with label  y=1 . The cross entropy loss function could easily be influenced by the problem of class imbalance between the foreground and background, which results in instability during one-stage the training processes. Therefore, the focal loss could be defined as:

(2) $$FL\left(p_{t}\right)=\;-\alpha_{t}{\left(1-\;p_{t}\right)}^{\gamma }\log\left(p_{t}\right)$$

where ${\left(1-\;p_{t}\right)}^{\gamma }$ is a modulating factor to the cross entropy loss defined in Equation (1), and γ≥0 serves as a tunable focusing parameter that adjusts the rate at which easy examples are down-weighted to reduce their loss contribution. Similarly, as in cross entropy loss Equation (1), $p_{t}$ represents the estimated probability for class 1. Moreover, α-balanced is a variant of the focal loss, where α is a weighting factor α ∈ [0,1] for class 1 and 1-α for class -1.

**Figure 2:**
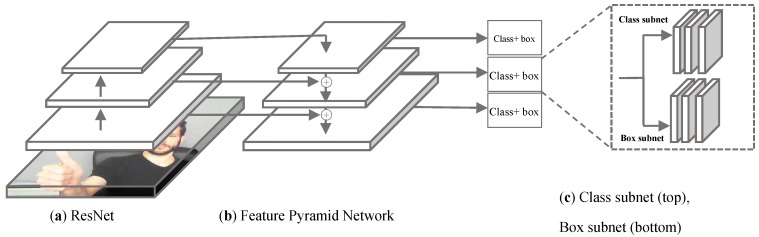
The proposed RetinaNet architecture contains three blocks: (**a**) A feedforward ResNet architecture [16] is used to generate a multi-scale convolutional feature pyramid (the encoder); (**b**) The feature pyramid network (decoder); (**c**) Two sub networks are used for box classification (top) and for anchors boxes regression (bottom).

We have adopted this solution for our hand detector. The system, which has been proposed for hand detection, is required to be fast and robust. Considering the tradeoff between speed and accuracy, we adopted a one-stage object detector for hand detection based on the RetinaNet architecture, as depicted in Figure 2 [21].

The proposed hand detector is composed of a backbone network, which is necessary for computing a convolutional feature map over the entire input image and two task-specific subnetworks for bounding-box classification and regression on the backbone’s output, shown in Figure 2.

The architecture adopts the encoder-decoder paradigm known as the feature pyramid network (FPN) [42], which is built on top of the feedforward ResNet architecture [16] to generate a rich, multi-scale convolutional feature pyramid in a fully convolutional fashion from a single resolution input image. Each layer of the pyramid can be used for detecting objects at different scales. FPN involves two bottom-up and top-down pathways, which are connected with lateral connections. The bottom-up pathway consists of a usual convolutional network for feature extraction (feature encoder). In our case, we chose the ResNet-50 architecture, as shown in Table 1. We chose this architecture as it possesses a good tradeoff between speed and accuracy [21,23]. In the proposed architecture, ResNet-50 is used without the top layers (average pool, fully-connected layer, softmax layer, see Table 1).

**Figure 3:**
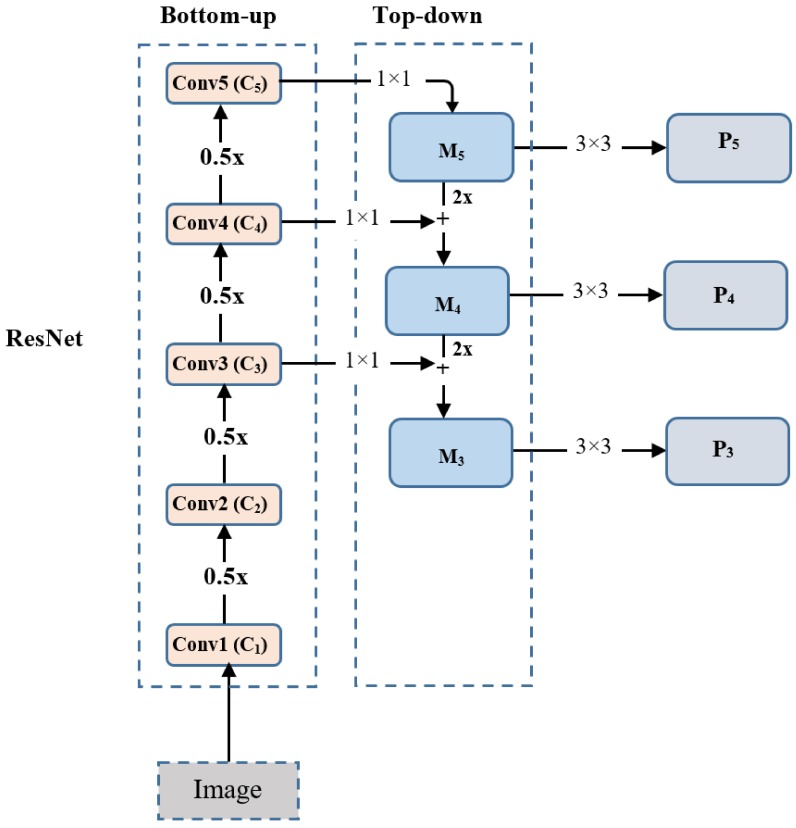
The architecture of the backbone network with ResNet-50 [16] and the feature pyramid network [42].

As shown in Figure 3, the bottom-up pathway is responsible for computing a feature hierarchy consisting of feature maps at different scales with a scaling step of 2. It is worth mentioning that some consecutive layers in ResNet may produce output maps of the same size, and we consider these layers at the same network level [16,42]. As we move up in ResNet-50, shown in Figure 3, the spatial dimension decreases by a factor of 0.5. The output of the last residual block is used as the reference set of feature maps and is denoted as $C_{i}\;$(i∈[1, 5]). Note that we do not use $C_{1}\;\mathrm{and}\;C_{2}$ in the pyramid because of their large memory footprint [21,42], as shown in Figure 3.

In contrast, the top-down pathway, shown in Figure 3, moves from the deepest layer of the network (last level of the bottom-up pathway) and progressively up-samples it, while adding in the transformed versions of higher-dimension features from the bottom-up pathway. Starting from the deepest layer of the bottom-up pathway, FPN applies a 1 × 1 convolutional layer to reduce the $C_{5}$ channel depth to 256 in order to create $M_{5}$, and then a 3 × 3 convolutional layer is used to generate $\;P_{5}$, which is the final feature map that is fed to the rest of the network for prediction. For each consecutive layer, the previous feature map is up-sampled by a factor of 2, using nearest neighbor upsampling. Then, the up-sampled feature map is added to the corresponding bottom-up feature map (which undergoes a 1 × 1 convolutional layer to reduce the channel dimensions) using an element-wise summation. A 3 × 3 convolutional layer is again applied to the merged feature map to generate the corresponding final feature map, which will be used for prediction. As in [21], five feature pyramid levels ($P_{3}\;to\;P_{7}$) are applied here, where we construct pyramid levels $P_{3}\;to\;P_{5}$ from the corresponding ResNet convolutional building block ($C_{3}\;to\;C_{5}$) using the aforementioned process. Differently, $P_{6}$ is computed using a 3 × 3 stride-2 convolutional layer on $C_{5}$, and $P_{7}$ is obtained by applying a rectified linear unit (ReLU) activation function, followed by a 3 × 3 stride-2 convolutional layer on $\;P_{6}$. It is worth mentioning that $\;P_{2}$ is not used for computational reasons [21], while $P_{7}$ is included to enhance large object detection.

As shown in Figure 2, the subnetwork called the classification subnetwork is responsible for predicting the probability of hand presence at each spatial position for each of the A anchors and K object classes. The subnetwork represents a small fully convolutional network (FCN) connected to each FPN level. The parameters of this subnetwork are thus shared through all pyramid levels. The input of this subnetwork is a feature map, with C channels taken from a given pyramid level. The subnetwork applies four 3 × 3 convolutional layers on the input feature map. Each convolutional layer has C filters followed by ReLU activations, followed by another 3 × 3 convolutional layer with KA filters. Finally, sigmoid function activations are connected to the output KA binary predictions for each spatial location. In our experiments, we used C = 256 and A = 9. The focal loss defined in Equation (2) is applied to the output of this subnetwork. Similar to the classification subnet, the box regression subnetwork is another small FCN, attached to each pyramid level, used for regressing the offset from each anchor box to an adjacent ground truth object, if such an object exists. It shares the same design as the classification subnetwork, except that its output is a vector of 4A linear outputs per spatial location, where A is the number of anchors. Although both the classification and box regression share a common structure, the parameters are not shared. For each of the A anchors per spatial location, these 4 outputs predict the relative offset between the anchor and the ground truth box [21]. The smooth L1 loss function (Equation (3)) is applied to the output of the box regression subnetwork as the loss function [35]:

(3) $$L_{1};smooth=\{\begin{array}{ll} \left|x\right|, & if\;\left|x\right|>\alpha \\ \frac{1}{\left|\alpha \right|}x^{2}, & else\;if\;\left|x\right|\leq \alpha \end{array}$$

where *α* is a hyperparameter that is usually set to 1 and *x* is the *L*_1_ distance between the predicted and ground truth vectors.

### 3.2. Lightweight CNN-Based Hand Gesture Recognition

**Figure 4:**
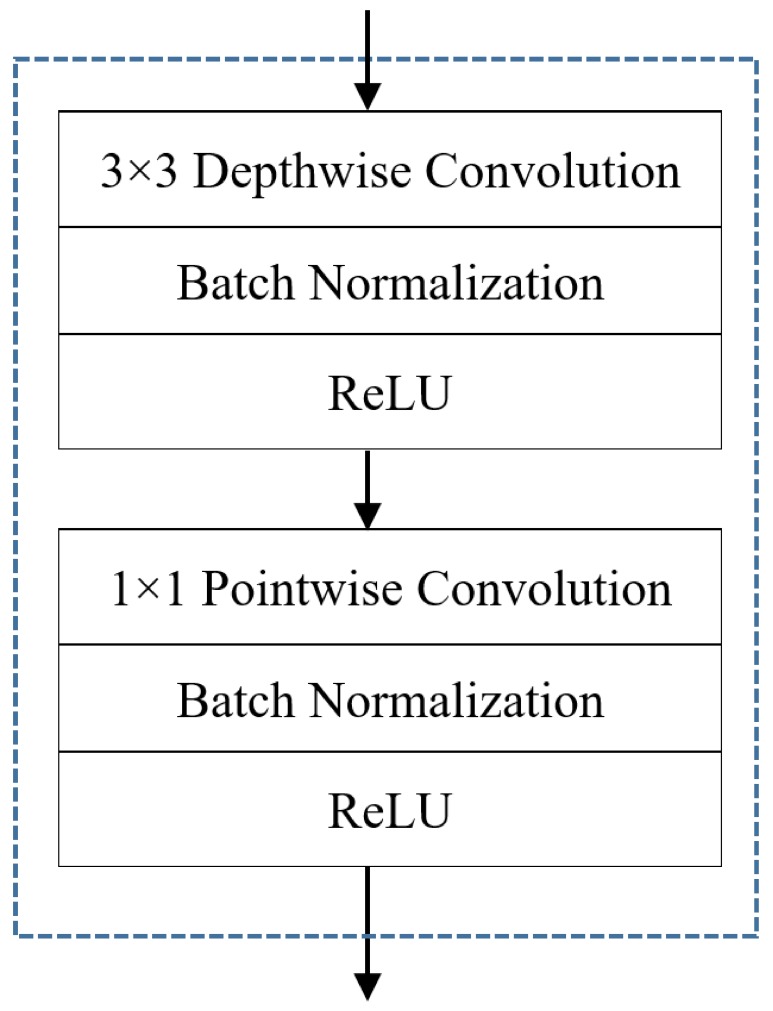
Illustration of the depthwise separable convolution block used in MobileNet [22].

Once the hand is detected, a CNN-based classifier is responsible for classifying the detected hand gesture into one of the predefined gesture classes. Here, the choice of the CNN architecture is critical and is usually motivated by a trade-off between speed and accuracy. In our application, we sought an architecture that achieves a good classification performance and speed while maintaining a minimal usage of memory and computational cost. In order to fulfill the resource-constrained conditions, we used the lightweight MobileNet [22] model as a gesture classifier in our architecture. MobileNet is designed for efficient inference in devices with limited processing capabilities. It utilizes depthwise separable convolutions, which factorize a standard convolution into a depthwise convolution and a 1 × 1 pointwise convolution, as shown in Figure 4, allowing us to create very small image classification models and effectively reduce both the computational cost and the number of parameters. The gesture classification stage takes a 64 × 64 cropped three channel image of the hand as a network input. It extracts discriminative features from the gesture entered and passes them to a softmax layer for gesture classification. The cross entropy (CE) loss function was employed on the output of this subnetwork.

To further reduce the number of parameters, MobileNet has also proposed two hyper-parameters (width multiplier and resolution multiplier) that efficiently tradeoff between latency and accuracy. The purpose of the width multiplier α (α ∈ [0,1]) is to thin out a network uniformly at each layer. Applying this multiplier produces a new smaller model with an acceptable trade-off between the desired latency and performance. A resolution multiplier ρ (ρ ∈ [0,1]) is applied to the input image to scale the input size of the image in order to produce a reduced computation neural network [22]. In our experiments, we used the baseline model hyperparameters by setting the width and resolution multipliers to one, and we implicitly rescaled the input image to 64 × 64, as the hand gesture occupies only a small part of the image.

## 4. Experiments and Discussions

Hand detection and gesture recognition are challenging tasks in unconstrained environments due to the deformable and articulated nature of hands. Therefore, we proposed a deep learning approach for detecting and recognizing static hand gestures in real life scenarios. In this section, we elaborately discuss all of the experiments performed to evaluate the robustness of the proposed approach.

### 4.1. Experimental Details

#### 4.1.1. Experimental Datasets

To evaluate the performance of the proposed architecture for both tasks (i.e., hand detection and gesture recognition), we performed experiments based on a two-stage training strategy, namely, training the hand detector and training the gesture recognition. In the first stage, we trained the first part of the architecture using our collected dataset. The dataset was divided into two subsets for training and validation, respectively. This part ensures the robustness of hand detection in any given situation. Subsequently, the ready hand detector was used to infer unlabeled data (hand gesture datasets). The second stage consists of training the lightweight CNN model for hand gesture recognition. The detailed description of the used datasets is given as follows:

1. Hand detection datasets: Due to the limited size of the existing standard datasets for hand detection, and in order to train our hand detector with sufficient data, we created a new dataset to use in our experiments. We have collected a combined dataset containing a total of 24,535 images and over 41,000 hand instances. To ensure the diversity of the data collected, the dataset combines samples from different datasets, (e.g., those from [12,24,44,45]) and other images sources. In addition, we are more interested in creating a realistic and diverse dataset in terms of viewpoints (first and third person views, etc.), the number of subjects involved, different indoor/outdoor environments, and diversity as well, in terms of the engaged hand activities (e.g., gesturing, playing, engaging in conversations, housework, etc.). It is worth mentioning that the collected dataset is not comprised of any samples from the datasets used later to evaluate the detector performance, neither those used in the gesture classification phase. The collected dataset was prepared and annotated manually with ground truth bounding-boxes. We divided the training dataset randomly into two subsets: 80% for training and the remaining 20% for the validation dataset, which was used to fine-tune the model during training. We have also used the same dataset for training in all our experiments to provide an unbiased comparison. To assess the detector performance and demonstrate the robustness and the generalization power of the hand detector, we evaluated the performance of the trained models on four different test datasets, namely, the Oxford [24], 5-signers [45], EgoHands [44], and Indian classical dance (ICD) datasets ([5]). Table 2 summarizes the characteristics of the datasets used for the training and testing of the hand detector.
2. Hand gesture recognition datasets: To train and evaluate the gesture recognition performance of our proposed architecture, two hand gesture datasets were chosen because they both have publicly available data with challenging data conditions, i.e., they contain a large amount of data with a different number of classes, as detailed below.
  - The LaRED dataset (Available at: http://mclab.citi.sinica.edu.tw/dataset/lared/lared.html) [46] is a large RGB-D hand gesture dataset that provides ~240,000 tuple images (color image, a depth image, and a mask of the hand region). To the best of our knowledge, this is the largest hand gestures dataset, with 81 different classes (27 hand gestures in 3 different rotations). The dataset has been collected using a short-range Intel depth camera. The classes have been recorded by 10 subjects (five males and five females), and each subject was asked to perform 300 gesture images per class, repeating the same hand gesture with slight movements. This large volume of labeled data is the best-suited set of data to develop and train deep learning algorithms for practical applications. Furthermore, this dataset is extensible, since it comes with the software used to record and inspect the dataset, allowing future users to increase the dataset size by adding more subjects/gestures in the future. For the sake of our needs, we used only the color images of VGA resolution and omitted the rest of the dataset. Following the baseline approach [46], we have divided the entire dataset into two disjoint subsets, i.e., those used for training and testing. The test subset contains 10% of the total data, while the remaining is used during the training process.
  - The TinyHands (In the original paper, they have not named this dataset. Therefore, we call it TinyHands as an abbreviation) gesture dataset (Available at: https://sites.google.com/view/handgesturedb/home): This dataset [47] has been captured in two distinct setup environments. Half of the dataset has been recorded with a simple background and the rest with a complex background. The complex background undergoes various illumination conditions with a highly cluttered environment. In the dataset, the gestures are performed in different locations in the image and hands occupy only a small region of the image (about 10% of the whole image in pixels). There were forty participants involved to collect this dataset. Each participant was asked to make seven different gestures, and about 1400 frames compose every instantiation of one gesture. Following the baseline approach [47], we have used a subject independent approach, where subjects who appear in the testing set are totally different from the training set. We have employed a cross-validation strategy with four repetitions, in which each repetition uses 25 subjects for training, 5 subjects for validation, and 10 subjects for testing. Table 3 summarizes the characteristics of the datasets used for hand gesture recognition.

#### 4.1.2. Evaluation Metrics

To assess the performance of our model, the evaluation of our proposed model has been done in two contexts, namely, detecting hands of any type, then detecting and recognizing hand gestures. For hand detection, most of the existing hand detection datasets consider average precision (AP) to evaluate the performance of the hand detector. Assigning a bounding box as a true positive for the hand hand depends on the PASCAL VOC criteria [48] for scoring detections (that the threshold of intersection over union (IOU) between detected ($B_{DET}$) and ground truth ($B_{GT}$) bounding boxes is at least 0.5). We report the average precision (AP), average recall (AR), and F_1_ score for each of the test datasets. In our experiments, we do not consider true negatives (i.e., ground truth data of non-hand regions), as we are interested only in whether the detected hand is correct or not. We have only calculated true positives (TP), false positives (FP), and false negatives (FN) to obtain precision, recall, F_1_ score, and accuracy (Equations (4)–(8)). We calculated the abovementioned metrics as follows:

(4) $$Precision=\;\frac{\mathrm{TP}}{\mathrm{TP}\;+\;\mathrm{FP}}$$

(5) $$\;Recal\;=\;\frac{TP}{TP\;+\;FN}\;$$

(6) $$\;F_{1}\;score\;=\;2\;\times \;\frac{Precision\;\times \;Recal}{Precision\;+\;Recal}\;$$

(7) $$\;IoU\left(B_{GT},B_{DET}\right)\;=\;\frac{B_{GT}\cap B_{DET}}{B_{GT}\cup B_{DET}}\;$$

(8) $$\;Accuracy\;=\;\frac{TP}{TP\;+\;FP\;+\;FN}\;$$

As shown in Section 4.2.1, we have considered the precision-recall (PR) curve to depict the tradeoff between precision and recall. We have also evaluated the efficiency of our model by comparing its average precision in terms of what is obtained with other methods. To demonstrate the performance of the whole architecture, evaluation was done using hand gesture datasets, and then the average recognition accuracy was calculated as an evaluation metric. For multi-class gesture classification, we have reported the confusion matrix for each dataset, shown in Section 4.2.2, where rows represent ground truth annotations and columns represent the inferred classes. The average accuracy was then calculated as the mean of the main diagonal of the confusion matrix.

#### 4.1.3. Implementation Details

Our proposed system has been implemented with Keras [49], the deep learning toolbox for Python, using Tensorflow [50] as the backend. We performed the training using the NVIDIA Tesla K80 graphics card with an Intel Xeon 2.30 GHz CPU and 12 GB of RAM. Firstly, we started by training the hand detector on our collected dataset. Following the common practice, the training dataset was divided into 80% for the training set and 20% for the validation set, which was used for hyperparameter optimization. The ResNet-50-FPN backbone was initialized with pre-trained weights on the COCO detection benchmark [51]. Then, we used a method for stochastic optimization (Adam) with a minibatch size of one (the small minibatch size was chosen for memory reasons). We empirically initialized the learning rate at 10^−5^ and decreased it by a factor of 0.1 when the training loss plateaued for more than 10 consecutive epochs [16]. To avoid overfitting and find the optimal training time, a regularization technique called early stopping was adopted, in which the performance on the validation set was monitored. This early stopping occurred at epoch 150. In addition, we have employed data augmentation as another technique to avoid overfitting, by introducing more training data and achieving better performance while training the deep learning models. We implemented on the fly random horizontal image flipping as the only form of data augmentation, as it is one of the most widely used data augmentation techniques among previous research [21,23]. Subsequently, the ready hand detector was used to infer unlabeled data for training the hand gesture recognition model. To train the lightweight CNN model for gesture classification, the Adam optimizer was also used to train the model, with a minibatch size of 32. The same early stopping technique was used to control the training time. For this model, the initial learning rate was empirically set to 10^−3^ and was decreased by a factor of 0.1 when the training loss plateaued for more than 10 epochs.

### 4.2. Experimental Results

#### 4.2.1. Hand Detection Results

Hand detection is indispensable in many human interaction systems, however, hand detection is an extremely challenging problem due to the deformable and articulated nature of hands. As well as the different contributing factors to this complexity, including cluttered backgrounds and varying illumination conditions also contribute here. In addition, hands always occupy a small region within an image, which results in a large background area. The robustness of the hand detection algorithm should be demonstrated on images with varying degrees of complexity in terms of clutter in the background. Therefore, we tested our proposed approach for hand detection on different publicly available datasets, including the Oxford hand dataset [24], 5-signers dataset [45], EgoHands dataset [44], and the Indian classical dance (ICD) dataset [5]. Table 4 and Table 5 summarize the results of the hand detection task using our proposed detector with Resnet-50-FPN as a backbone. Table 4 shows the results according to each test dataset. We estimated the average precision (AP), average recall (AR), and F1 score for each test dataset.

Since our approach firstly aims to ensure the accurate detection of hands in varying challenging scenarios, our method was evaluated on different datasets that had different levels of complexity. As shown in Table 4, our approach achieved the best results with the 5-signers and EgoHands dataset, concerning both the average precision (which indicates the proportion of correctly detected hands out of all detected regions) and average recall (which indicates the proportion of hands detected out of all of the hands that exist). The performance of the hand detector on the ICD dataset trails those results, with 85.5% for the AP and 67.9% for the AR. However, when the detector was tested on the challenging Oxford dataset, the performance dropped. This is likely because of much smaller, low resolution, and incomplete hands in images. Nevertheless, these results outperformed other approaches, as seen in Table 5.

To compare our approach with previous approaches, Table 5 summarizes the performance (average precision) of our proposed approach and shows comparisons with other typical approaches, including R-CNN [5,14], R-CNN and skin [5], Faster R-CNN [5,15], Faster R-CNN and skin [5], the approach from Bambach et al. [44], the multiple proposals approach, [24] and the approach from Deng et al. [36].

Compared with the baseline method [24] and other methods [5,14,15,36] on the well-known and widely used Oxford dataset for hand detection, our approach has achieved an average precision of 72.1%, which is higher than the best AP obtained in [36] by 14%. Despite the challenging task of detecting hands in this dataset (due to severe occlusion and the small sizes of the hands in some images), the hands can still be correctly detected. This proves the efficiency of our proposed detector to tackle challenging images where hands are small and blurred. Similarly, our hand detector outperforms other methods on the 5-signers dataset, with an average precision of 97.9%, surpassing the present state-of-the-art method [5] by 0.63%. The 5-signers dataset contains humans performing gestures from news sequences, where the spatial resolution of hands in the images is less, with tiny and sometimes overlapping hands.

Bambach et al. [44] created the EgoHands dataset for hand detection and presented a method based on CNN region sampling. Their baseline method obtained an 80.7% average precision value. However, as shown in Table 5, the recent work in [5] shows a good performance when combining a two-stage detector (Faster R-CNN [15] and R-CNN [14]) with skin segmentation on the EgoHands [44] dataset. Their approach has achieved state-of-the-art performance with the EgoHands test datasets, with 96% average precision. They took advantage of skin detection technique, which significantly reduces the occurrence of false positives. However, it failed to detect hands in highly cluttered and dynamic light environments (i.e., in the case of the Oxford [24] and ICD datasets [5]). Nevertheless, our approach outperforms [5] in the Oxford, 5-signers, and ICD test datasets, and achieves competitive performance with the EgoHands dataset. The reason for this is that the method in [5] benefits a lot from the eliminated non-skin areas in the second stage of their approach, due to the fact that images in the EgoHands dataset [44] are from an egocentric view and have been captured using Google Glass, where images are sufficiently large enough and the hands are in always in the foreground.

The proposed approach surpasses [5] other approaches in terms of the ICD dataset performance by a large margin, which demonstrates the adequacy of the hand detector to locate hands in unconstrained environments, where images suffer from complexities in costumes, make-up, cluttered environments, people in background, etc.

As shown in Table 5, it can be seen that the detection performance varies according to each dataset. This is mainly due to the different characteristics of each dataset in terms of the acquisition environments, level of complexity, and the quality of the images. Nevertheless, the detection performance shows a significant improvement over previous approaches. These improved results are due to the two main components that comprise our hand detector: The feature pyramid network (FPN), which allows a multi-scale prediction, and thus hand detection can be made no matter its scale, especially for small hands; and the focal loss, which addresses the imbalance problem of the foreground and background objects during the training of the detector, which results in boosting the detection performance. These factors, along with training the detector with a large collection of widely-varying data, have contributed to obtaining the currently presented reliable hand detector.

**Figure 5:**
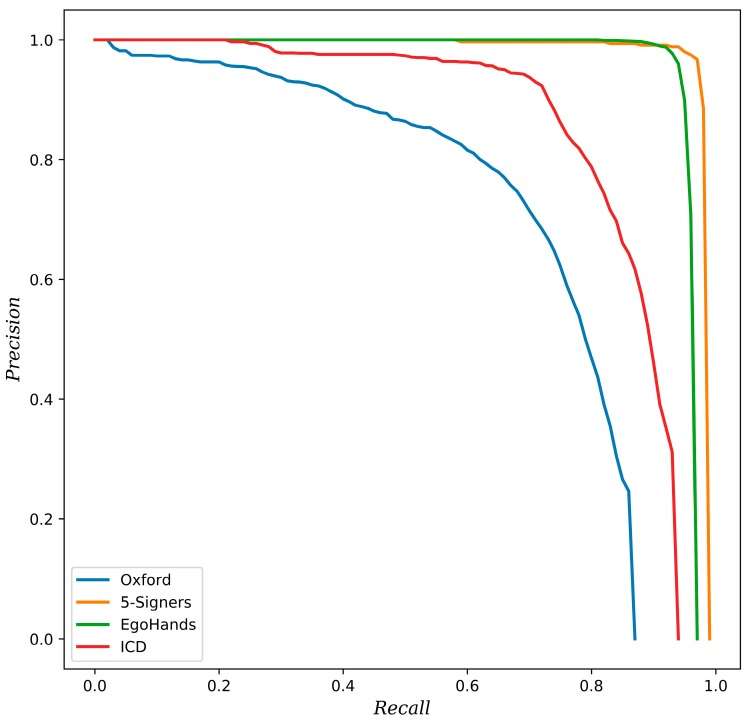
Precision-recall (PR) curves for different datasets.

Figure 5 shows the precision-recall (PR) curve obtained using the proposed approach for the Oxford [24], 5-signers [45], EgoHands [44], and ICD [5] datasets. The precision-recall (PR) curve depicts the tradeoff between the sensitivity and precision, where a good detector will balance the precision and recall, so that the corresponding area under Precision-Recall Curve should be large, i.e., a high value of AP.

#### 4.2.2. Hand Gesture Recognition Results

**Figure 6:**
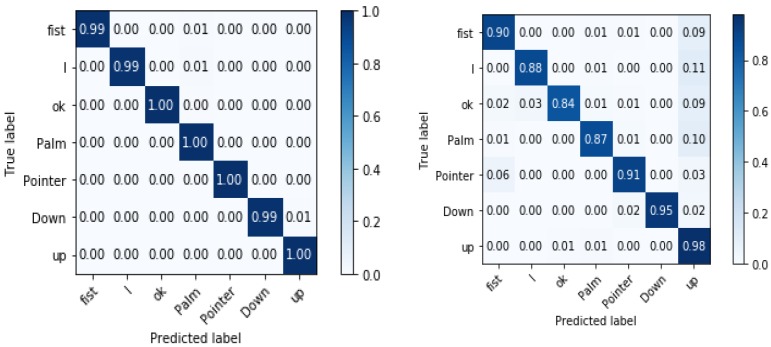
The confusion matrices obtained using the proposed approach on the TinyHands dataset: Simple background (**left**) and complex background (**right**).

In this section, the whole system architecture for hand gesture detection and recognition is evaluated using the LaRED Benchmark dataset [46] and the TinyHands dataset [47]. Here, the evaluation metrics are the average accuracy and confusion metrics, as given in Section 4.1.2. In our experiments, the gesture recognition model is the only part which needs to be retrained for a different hand gesture dataset each time. The input images go through the architecture to extract hand regions, in which these are later processed by the hand gesture recognition model for gesture recognition. As shown in Table 6, we have summarized the gesture recognition results obtained on the LaRED [46] and simple and complex TinyHands [47] test subsets. It is important to mention that the results reported are the average of all classes in each subset. Our approach achieves the best results for both the LaRED and simple TinyHands datasets, with 97.25% and 99% as overall accuracies, respectively. We have also obtained good results for the complex TinyHands dataset, with an overall accuracy of 90.43%, which is considerably high, considering the small size of the hand and the complexity of the surrounding environment. As shown in Figure 6 (right), it can be observed that some classes have confusion with other classes, due to the low interclass variance.

**Figure 7:**
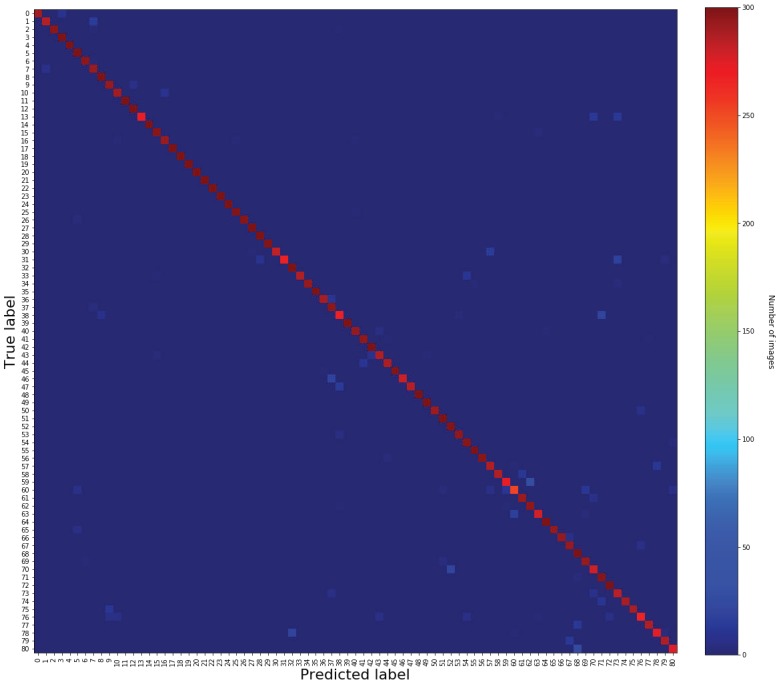
The confusion matrix obtained using the proposed approach on the LaRED dataset. For better visualization, scores of the 81 classes are scaled and represented in different colors (best viewed in color).

Besides, we have compared the results obtained with the previous methods in Table 7 and Table 8. We have achieved the best results and surpassed other methods in terms of accuracy by a large margin. For better visualization of per class accuracy, we also provide a confusion matrix for each of the test subsets, shown in Figure 6 and Figure 7.

We compared our method with the previous approaches on the TinyHands dataset, where the comparison is presented in Table 7. Our proposed method outperforms all the other methods and surpasses the best results obtained for the simple and complex subsets by 1.9% and 5.13%, respectively. The performance comparison with the previous methods on LaRED dataset is presented in Table 8. We have also found that our proposed architecture outperforms other methods and surpasses the best results obtained in [52] by 8.53%, which is high, considering the small interclass variances and large number of gesture classes.

As shown in Table 7 and Table 8, the results obtained show a significant improvement in recognition accuracy. This improvement is due to the efficient recognition performance of the proposed lightweight CNN and the accurate hand detection which results in higher classification accuracy (e.g., the case of the complex subset), since the stage of hand detection helps to focus on the hand areas and eliminate the complex background.

**Table 7:** Comparison of the accuracies obtained on TinyHands gesture datasets with the previous methods.

Method	Simple (%)	Complex (%)
AlexNet [47,53]	86.30	69.40
VGG19 [47,54]	96.20	77.60
Baseline Network [47]	97.10	85.30
Our Method	99.00	90.43

**Table 8:** Comparison of the accuracies obtained on LaRED gesture datasets with the previous methods.

Method	Accuracy (%)
Deep belief networks (DBN) [52]	66.13
Restricted Boltzmann machines (RBM) [52]	72.95
Baseline Network [46]	74.55
Stacked autoencoders (SAE) [52]	81.09
CNN [52]	88.72
Our Method	97.25

#### 4.2.3. Processing Speed of the Proposed Architecture

The time consummation of our architecture during the inference procedure has been explored. Here, frames per second (FPS) has been used as the evaluation metric to evaluate the speed performance of the proposed architecture. Table 9 presents the time efficiency at five different scales (400–800 pixels). As shown in Table 9, small input image resolutions yield higher speeds. However, this may degrade the detection accuracy, because high resolution inputs allow for small objects to be resolved, which results in better detection accuracy [23]. Nevertheless, our proposed approach achieves fast results in terms of processing time, with approximately 12 FPS on the GPU, which can meet the basic needs for gestural applications.

### 4.3. Discussion

We have proposed an effective deep learning-based composite network architecture to jointly detect hands and recognize static hand gestures. The proposed architecture detects hands from an RGB input image using a one-stage object detector based on RetinaNet [21] and passes the detected hand through a lightweight CNN to recognize hand gestures. We have conducted several experiments to evaluate the proposed architecture on different challenging datasets for hand detection and gesture recognition. The results of our proposed method are elaborated in Section 4.2.1 and Section 4.2.2.

**Figure 8:**
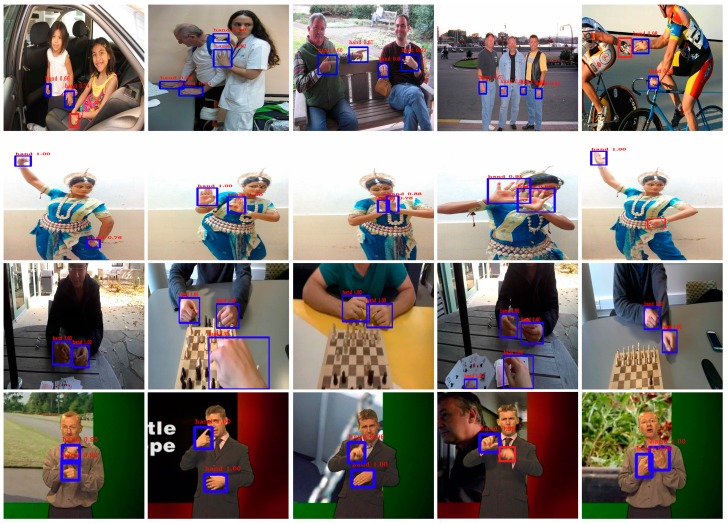
Some examples of hand detection results for different hand detection datasets. The first row contains images from the Oxford test dataset [24]. The second row contains images from the ICD dataset [5]. The third row contains images from the EgoHands dataset [44]. The fourth row contains images from the 5-signers dataset [45]. The true hand regions are detected and marked with blue bounding boxes, hand labels, and detection scores, whereas the misclassification hand regions are marked with red bounding boxes (best viewed in color).

As shown in Table 4 and Table 5, our approach has achieved good results for different challenging datasets, which demonstrate the effectiveness of our approach for robust hand detection and gesture recognition. We have also compared our results with previous methods for both hand detection and gesture recognition tasks, as seen in Table 5, Table 7 and Table 8. As shown in Table 5, our method presents a higher average precision (AP) for three hand detection datasets (Oxford [24], 5-signers [45], and ICD [5]) over the other methods, while maintaining competitive performance for the EgoHands dataset [40]. Despite the challenging images taken in practice, which are usually small and have low resolution, as shown in Figure 8 (first row), we have achieved a 14% improvement in average precision for the Oxford dataset over the previous best result, obtained in [36]. This high performance is due to the two main building blocks of our approach, i.e., the FPN to extract the multi-scale semantic features, and focal loss to deal with the class imbalance and unfair contribution of hard and easy examples to the loss.

The results obtained for the ICD [5] dataset also prove the superiority of our hand detector over the previous state-of-the-art method [5], where it achieved a 50.17% higher average precision (AP). The method in [5] relies on a two-stage architecture, in which a skin detection algorithm is used to improve the detection results obtained from two state-of-the-art object detectors (R-CNN [14] and Faster R-CNN [15]). Their method failed to detect hands in highly cluttered and light changing environments, which is the case of the ICD dataset, where hands suffer from high occlusion and changing lighting conditions, as well as the blurring of skin color due to the fast movement of hands in recorded videos, as can be seen in Figure 8 (second row).

Similarly, on the 5-signers dataset, our method achieved improved performance than in previous works, despite the fact that this dataset has been sampled from news sequences, resulting in low-resolution, blurred, and sometimes overlapping hands. We also gained comparable performance (93.1%) for the EgoHands dataset over the current best results [5], since the method in [5] had been tuned to detect hands with clear skin color, which is the case for the EgoHands dataset, where the images are large enough and the hands are in the foreground.

In fact, there are many factors that should be considered, all of which have great influence on the performance of hand detection algorithms, such as the hand variations, level of occlusion, resolution, lighting conditions, and variation in shape and viewpoint, along with the quality of the image captured. As shown earlier, the proposed hand detector achieves the best performance in several challenging datasets. This is due to the careful design of different blocks of RetinaNet, i.e., the FPN to generate the multi-scale semantic feature and focal loss to tackle the problem of class imbalance and the unfair contribution of hard and easy examples to the loss, which has resulted in a high performance detector with an excellent trade-off between accuracy, speed, and complexity. In Figure 8, we show some qualitative detection examples of our method from different hand detection datasets, depicting the complexity of hand detection in various scenarios.

**Figure 9:**
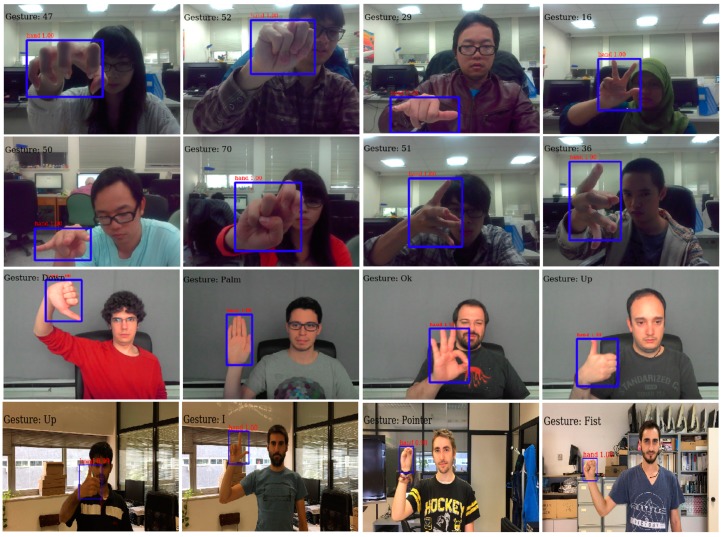
Some examples of hand detection and recognition results using our proposed architecture on different datasets. The first row contains images from the LaRED dataset [46]. The second and the third rows contain images from the simple and complex TinyHands datasets [47], respectively (best viewed in color).

To demonstrate the performance of the whole architecture on both tasks (detection and recognition), we used two hand gesture datasets, namely, LaRED [46] and TinyHands [47]. As shown in Section 4.2.2., our method achieves a higher recognition accuracy for both datasets than the other methods, regardless of the gestural vocabulary size or the complex surrounding conditions. The proposed architecture can robustly robustly seven gestures from the TinyHands dataset and up to 81 gestures from the LaRED dataset. Figure 6 and Figure 7 show the confusion matrices for both datasets. It is also worth mentioning that the system detects one gesture for each image. The system extracts the hand with the highest detection score and passes it through the gestural recognition stage for gesture recognition. In Figure 9, we have shown some qualitative results of our proposed architecture on test images from LaRED and TinyHands datasets, in which both the hand detection bounding boxes and gesture recognition results are shown. As these results show, our proposed architecture can handle different scales of hands, shapes, different illumination conditions, and recognize gestures in different complex scenarios. According to experimental results shown in Table 7 and Table 8, we conclude that accurate hand detection enhances the performance of the gesture recognition system with fast processing, which in turn enables accurate human-machine interaction.

## 5. Conclusions

Hand detection and gesture recognition are crucial tasks for many interactive applications. Most previous works have attempted to solve the problem as separated tasks. Moreover, these tasks are challenging, due to the deformable and articulated nature of hands, and the potential for cluttered environments. In this paper, we proposed a deep learning network architecture, which aims to jointly detect and recognize hand gestures. The proposed architecture is based on a one-stage dense object detector for hand detection and a lightweight CNN network for gesture classification. Experiments were carried out on various hand detection and hand gesture recognition benchmarks to demonstrate the robustness and effectiveness of our approach. In addition, our architecture was trained and tested with datasets taken under various acquisition conditions to ensure the generalization ability of the trained model. Using our architecture, we achieved state-of-the-art performance and outperformed the previous approaches when using the Oxford, 5-signers, and ICD datasets as samples for hand detection. In addition, we obtained comparable results with the EgoHands dataset. We also tested our approach on two challenging datasets for hand gesture recognition and achieved excellent results in classifying gestures. Future work may investigate other CNN networks as a backbone for one-stage hand detection, and focus particularly on lightweight architectures to build a system for embedded vision applications. Despite the fact that we have focused on recognizing static hand gestures, our architecture can be extended to support dynamic hand gesture recognition by considering the temporal aspect of the gesture. We also intend to examine different solutions for multi-users interactions, where many users can interact simultaneously with the system. In addition, we intend to increase the size of our dataset with more annotated data for hand detection and gesture recognition.

## Figures and Tables

**Table 1:** The architecture of ResNet-50 [12] used in the backbone network. Batch normalization (BN) [43] is used after each layer and right before the rectified linear unit (ReLU) activation function. 2/1* means 2 at the first iteration and 1 from the second iteration.

Layer Name	Output Size	Kernel Size	Number of Filters	Stride	Number of Iterations
conv1	300 × 300	7 × 7	64	2	1
Maxpool		3 × 3	1	2	1
Conv2_x	150 × 150	$\;\left[\begin{array}{c} 1\times 1\;\\ 3\times 3\;\\ 1\times 1 \end{array}\right]\;$	$\;\begin{array}{c} 64\\ 64\\ 256 \end{array}\;$	$\;\begin{array}{c} 2/1^{*}\\ 1\\ 1 \end{array}\;$	x 3
Conv3_x	75 × 75	$\;\left[\begin{array}{c} 1\times 1\;\\ 3\times 3\;\\ 1\times 1 \end{array}\right]\;$	$\;\begin{array}{c} 128\\ 128\\ 512 \end{array}\;$	$\;\begin{array}{c} 2/1^{*}\\ 1\\ 1 \end{array}\;$	x 4
Conv4_x	38 × 38	$\;\left[\begin{array}{c} 1\times 1\;\\ 3\times 3\;\\ 1\times 1 \end{array}\right]\;$	$\;\begin{array}{c} 256\\ 256\\ 1024 \end{array}\;$	$\;\begin{array}{c} 2/1^{*}\\ 1\\ 1 \end{array}\;$	x 6
Conv5_x	19 × 19	$\;\left[\begin{array}{c} 1\times 1\;\\ 3\times 3\;\\ 1\times 1 \end{array}\right]\;$	$\;\begin{array}{c} 512\\ 512\\ 2048 \end{array}\;$	$\;\begin{array}{c} 2/1^{*}\\ 1\\ 1 \end{array}\;$	x 3

**Table 2:** Description of each hand detection dataset.

Dataset	Training (Images)	Testing (Images)	Number of Instances
Oxford	4069	823	13,049
5-Signers	3935	2000	8855
EgoHands	3600	800	15,053
ICD	-	675	1240
Our Collected	8633	-	9985

**Table 3:** Description of each hand gesture dataset.

Dataset	Number of Subjects	Number of Gestures	Resolution	Training Set	Testing Set
LaRED	10	81	640 × 480	~218,700	~24,300
TinyHands	40	7	1920 × 1080 640 × 480	~294,000	~98,000

**Table 4:** Results obtained using our proposed method on different hand detection datasets. AP and AR refer to (mean) average precision and average recall, respectively. ICD: Indian classical dance.

Dataset	AP (%)	AR (%)	F_1_ Score (%)
Oxford	72.1	45.1	54.9
5-signers	97.9	90.1	93.8
EgoHands	93.1	94.4	93.7
ICD	85.5	67.9	75.7

**Table 5:** Comparison of the average precision (AP) obtained on different hand detection datasets with previous methods. R-CNN: Region-convolutional neural network.

Method	Oxford	5-Signers	EgoHands	ICD
R-CNN [5,14]	31.23	95.56	57.27	25.69
R-CNN and skin [5]	49.51	97.27	92.96	35.33
Faster R-CNN [5,15]	14.22	29.03	50.00	24.39
Faster R-CNN and Skin [5]	31.12	69.00	**96.00**	31.88
Bambach et al. [44]	N/A	N/A	84.20	N/A
Multiple proposals [24]	48.20	76.67	N/A	N/A
Deng et al. [36]	58.10	N/A	77.10	N/A
**Ours**	**72.10**	**97.90**	**93.10**	**85.50**

**Table 6:** Results obtained using our proposed method on different hand gesture datasets.

Dataset	Precision (%)	Recall (%)	F1 Score (%)
LaRED Dataset	97.33	97.25	97.29
TinyHands (Simple)	99.48	99.48	99.48
TinyHands (Complex)	91.36	90.41	90.88

**Table 9:** Comparison of inference time per image according to the image input resolution.

**Input Resolution**	400	500	600	700	800
**Frames Per Second (FPS)**	12.05	10.76	8.76	7.59	6.44
